# Global invasion network of the brown marmorated stink bug, *Halyomorpha halys*

**DOI:** 10.1038/s41598-017-10315-z

**Published:** 2017-08-29

**Authors:** Rafael E. Valentin, Anne L. Nielsen, Nik G. Wiman, Doo-Hyung Lee, Dina M. Fonseca

**Affiliations:** 1 Department of Ecology, Evolution & Natural Resources, Rutgers University, 14 College Farm Rd., New Brunswick, NJ 08901 USA; 2 Department of Entomology, Rutgers University, 93 Lipman Drive, New Brunswick, NJ 08901 USA; 3 Department of Horticulture, Oregon State University, 4017 ALS Building, Corvallis, OR 97330 USA; 4 Department of Life Sciences, Gachon University, Seongnam-si, Gyeonggi-do South Korea

## Abstract

Human mediated transportation into novel habitats is a prerequisite for the establishment of non-native species that become invasive, so knowledge of common sources may allow prevention. The brown marmorated stink bug (BMSB, *Halyomorpha halys*) is an East Asian species now established across North America and Europe, that in the Eastern United States of America (US) and Italy is causing significant economic losses to agriculture. After US populations were shown to originate from Northern China, others have tried to source BMSB populations now in Canada, Switzerland, Italy, France, Greece, and Hungary. Due to selection of different molecular markers, however, integrating all the datasets to obtain a broader picture of BMSB’s expansion has been difficult. To address this limitation we focused on a single locus, the barcode region in the cytochrome oxidase I mitochondrial gene, and analyzed representative BMSB samples from across its current global range using an Approximate Bayesian Computation approach. We found that China is the likely source of most non-native populations, with at least four separate introductions in North America and three in Europe. Additionally, we found evidence of one bridgehead event: a likely Eastern US source for the central Italy populations that interestingly share enhanced pest status.

## Introduction

Over the past few decades the introduction of non-native species, which are species that have been moved by human action into novel habitats beyond their natural geographic range^1^, has increased rapidly and on a global scale^2, 3^. However, in order for any non-native species to become established it must first successfully navigate the first phase of the invasion process, which is transportation into a novel habitat^1, 3^. Inadvertent human-mediated transportation of organisms often occurs due to global trade of goods^4^. Therefore, the physical and energetic limitations of long distance dispersal are bypassed and can result in unintended rapid and widespread establishment of non-native species^5, 6^. If non-natives become destructive or dangerous in their novel habitats, and spread rapidly beyond their introduced location, they are considered invasive^1^. Prior to becoming invasive, newly introduced non-native species can go unnoticed for some time until populations reach levels that result in significant economic impacts and high management costs^7, 8^. This danger is inflated for countries that trade regularly, as trade has been positively correlated with introduction rates of non-native species^3, 9^. Research on invasive species has led to recommendations that preventive actions be taken to limit the impacts of new invaders^10^, such as a better understanding of the source and likelihood of introductions, followed by novel strategies that allow early detection of incipient non-native populations^11, 12^.

Approaches to identify the source of an invasive species often make use of genetic methods that compare haplotypes or allele frequencies from potential native ranges with those in the introduced population(s)^13^. While that strategy works when there are clear genetic discontinuities among the native populations being evaluated, it can still be difficult to assign formal probabilities to alternatives (e.g. whether the introduced population came from a native source or secondarily from another introduced population (i.e. bridgehead effect))^9, 13, 14^. This is especially the case when the analysis is based on a single or a few genetic loci, which has been common in the analyses of non-model organisms with worldwide distributions^15–18^. Approximate Bayesian Computation (ABC) is a statistically robust Bayesian analysis that allows the direct comparison of multiple introduction hypotheses (known as scenarios) providing distinct likelihoods for each. ABC performs inference computations under a Bayesian framework^13, 14, 19^ that takes into consideration putative evolutionary histories, or in this case introduction histories, by quantifying support for modeled scenarios given the data provided. ABC accomplishes this by generating simulated data, known as pseudo-observed datasets (PODs), and randomly selecting (with replacement) the PODs closest to the observed data (i.e. the genetic data collected) by a Euclidean distance measure. These PODs then have relative posterior probabilities calculated via a logistic regression estimate, which allows the user to determine the most probable invasion scenario^20^. Of course, ABC does have its drawbacks. First, the computational time and power necessary to run moderately complex invasion models can be fairly demanding, at times taking several days to complete one analysis. Second, the scenarios to be evaluated are created by the modeler, and can be subject to bias unless all possible alternative hypotheses are included. Finally, the data must be of sufficient quality to address the desired questions being modeled in the scenario. Without appropriate data, estimates can be biased and inappropriate conclusions can be drawn. When correctly executed, however, ABC is an excellent analysis method for determining the most probable invasion pathways of unintended introductions^13^.

Here we incorporated an ABC approach to unravel the pathways of the worldwide expansion of the brown marmorated stink bug (BMSB; *Halyomorpha halys* (Stål)). BMSB is native to Northeast Asia, but non-native populations of BMSB were first detected in the United States of America (US hereafter) in Allentown, Pennsylvania in 1996^21^. Since then the species has been detected in at least 40 US states, Canada, and several European countries^22–24^. BMSB can cause significant damage both to agricultural crops and ornamental plants^25^, such as the documented damage to tree fruits in New Jersey and Pennsylvania in 2006 and 2007^26^ and WV and MD in 2010 and 2011^27^. BMSB feeding injury has resulted in significant economic impacts to growers; a one-year loss in excess of 37 million USD across the mid-Atlantic in apples alone, as well as 100% losses to peaches in Maryland^25^, and 60–90% losses of peaches in New Jersey (the 4^th^ national peach producer)^28^ during a population outbreak in 2010. Since the outbreak in 2010, established BMSB populations have been detected as far south as Georgia and as far west as Michigan, which experienced high populations in 2016 and injury in apples (J Wilson personal communication); and in the Pacific Northwest, specifically Oregon and Washington, where injury to hazelnuts and small fruits has been documented^29, 30^. To evaluate possible BMSB source populations, Xu *et al*.^31^ analyzed genetic variation at two mitochondrial loci in US specimens collected between 2004–2008, as well as in specimens from several populations across the native range of China, Republic of Korea, and Japan. They found best match to populations from the northern region of China, around Beijing, and low mtDNA haplotype diversity in the US populations relative to the native range, possibly indicating a single introduction of a small number of individuals^31^.

In Europe, there are currently known established BMSB populations in Switzerland, Italy, France, Greece, Hungary, Serbia, and Romania^22–24, 32, 33^, with very recent detections also in Bulgaria, Russia, Georgia, and the Autonomous Region of Abkhazia (ARA)^34, 35^. The first detection of BMSB in Europe was in Zurich, Switzerland in 2007^36^, and soon after it was collected in several locations throughout Switzerland^37^. To find the source population(s), and determine the invasion pathways, genetic analyses were carried out in Switzerland, Italy, France, Greece, and Hungary using mtDNA^38–40^. The likely source(s) for many of the established European populations was not determined due to insufficient power of the analyses to match or reject the few native populations examined^38, 39^. Of note, Cesari *et al*.^40^ hypothesized that BMSBs in Lombardy, Italy were likely the result of southward spread from Switzerland (either natural or human mediated), while the population in Emilia-Romagna, Italy was an independent introduction into Italy possibly from the US^40^. Again, due to insufficient samples from the native range, the authors were unable to determine the likely source.

To address the limitations of the individual studies and provide an updated analysis of the worldwide expansion of BMSB, we developed a meta-analysis using existing data^31, 38–40^ and an expanded sampling in the US and native ranges. By combining these datasets, we aimed to shore the power of the analyses and increase the likelihood of ascertaining the source(s) of all non-native populations. We therefore amassed and analyzed the largest BMSB dataset to date, with more than 900 individual DNA sequences (both existing and newly generated) including 214 from its native range in China, the Republic of Korea, and Japan. Although we are using a single mtDNA locus, we make use of ABC as our analysis framework to reach robust conclusions about invasion pathways of this global pest species.

## Results

### Sequence data and haplotypes

We amplified and sequenced 685 bp of cytochrome oxidase I (CO1) for 110 of the 139 specimens examined by Xu *et al*.^31^, as well an additional 80 specimens collected in 2016 in the US (48), Japan (28), and the Republic of Korea (4). Combined with preexisting data from the European studies, we amassed a total of 916 individual DNA sequences for our global dataset (Table 1). In the pre-2008 specimens from the US we found one new haplotype (H7), previously unreported, likely because Xu *et al*.^31^ only sequenced the CO1 locus from four specimens ^31^. We found this new haplotype was restricted to California, and all remaining specimens across the Eastern US had the one haplotype (H1) previously reported (Table 1). Among the post-2008 US specimens sequenced all eastern specimens had the same haplotype (H1), while the western specimens again displayed additional genetic variation (Table 1). The new specimens from the western states (i.e. California, Oregon, and Washington) had five different haplotypes, two of them being haplotypes we had previously seen (H1 and H3) but three were new for the US (H7, H23, and H47).

**Table 1:** Illustration of the collections from (A.) native countries and (B.) non-native countries, as well as the specific localities within those countries, of BMSB with the number of specimens for each in parenthesis.

A.					B.		
Native specimens					Non-Native specimens		
Country	Locality	Haplotype			Country	Locality	Haplotype
(no. of specimens)	(no. of specimens)	(no. per haplotype)			(no. of specimens)	(no. of specimens)	(no. per haplotype)
China (158)	Heibei/Beijing (106)*	H1 (75)	H10 (1)	H17 (1)	United States (108)	New Jersey (14)	H1 (14)
		H2 (1)	H11 (1)	H18 (1)		Maryland (14)	H1 (14)
		H3 (14)	H12 (1)	H19 (1)		Georgia (6)	H1 (6)
		H4 (1)	H13 (3)	H20 (1)		Delaware (3)	H1 (3)
		H5 (1)	H14 (1)	H21 (1)		Massachusetts (2)	H1 (2)
		H6 (1)	H15 (1)			Mississipii (2)	H1 (2)
		H7 (1)	H16 (1)			New York (2)	H1 (2)
	Xi’an (6)	H1 (1)				Pennsylvania (6)	H1 (6)
		H33 (2)				Virginia (6)	H1 (6)
		H45 (1)				West Virginia (6)	H1 (6)
		H53 (2)				Ohio (4)	H1 (4)
	Nanjing (12)	H2 (5)	H26 (2)			Michigan (5)	H1 (5)
		H3 (1)	H34 (1)			California (13)	H1 (11)
		H22 (2)	H55 (1)				**H3 (1)**
	Anhui Prov. (9)	H1 (3)					H7 (1)
		H2 (1)				Oregon (21)	H1 (1)
		H3 (4)					**H3 (10)**
		H33 (1)					**H23 (1)**
	Fuzhou (7)	H1 (7)					**H47 (9)**
	Haidain (6)	H1 (5)				Washington (4)	**H3 (2)**
		H46 (1)					**H47 (2)**
	Hefei (7)	H22 (4)					
		H54 (3)			Canada (51)	Canada (51)*	H1 (49)
	Kunming (5)	H1 (1)					H6 (1)
		H17 (4)					H14 (1)
Japan (44)	Tsubuka (16)	H23 (1)	H45 (5)		Switzerland (223)	North Switzerland	H3 (164)
		H24 (1)	H56 (1)			(195)*	H8 (30)
		H27 (1)					H9 (1)
		H39 (1)				Lugano (28)	H1 (2)
		H41 (4)					H3 (25)
		H44 (2)					H8 (1)
	Yokote (13)	H27 (1)	H41 (1)	H50 (1)		Ticino (2)	H3 (2)
		H39 (2)	H42 (1)	H51 (3)			
		H40 (2)	H49 (1)	H57 (1)	Italy (40)	Emilia-Romagna (31)	H1 (31)
	Yuzawa (15)	H1 (1)	H40 (4)	H51 (5)		Lombardy (9)	H1 (1)
		H23 (1)	H43 (1)	H52 (1)			H3 (7)
		H39 (1)	H48 (1)				H8 (1)
Republic of	Yangpyeong (1)	H22 (1)			France (139)	Schiltigheim (139)	H1 (1)
Korea (12)	Suwon (4)	H2 (1)					H3 (136)
		H22 (1)					H8 (2)
		H25 (1)					
		H38 (1)			Hungary (84)	Budapest (84)	H1 (83)
	Chungcheong	H28 (1)					H3 (1)
	Province (4)	H35 (1)					
		H36 (1)			Greece (57)	Athens (57)	H1 (18)
		H37 (1)					H3 (4)
	East Seoul (2)	H22 (1)					H22 (2)
		H29 (1)					H30 (1)
	Anyang (1)	H22 (1)					H31 (1)
							H32 (8)
							H33 (23)

Haplotypes shown are from the CO1 locus, with the number of specimens representing each haplotype in parenthesis as well. Locations marked with an asterisk indicate a lack of specific location data per haplotype. Haplotypes in bold indicate new haplotypes found in the US post-2008. We inserted Haplotypes H24 through H29, when we realized that previous studies^38^ jumped from H23 to H30.

### Data analysis

**Figure 1:**
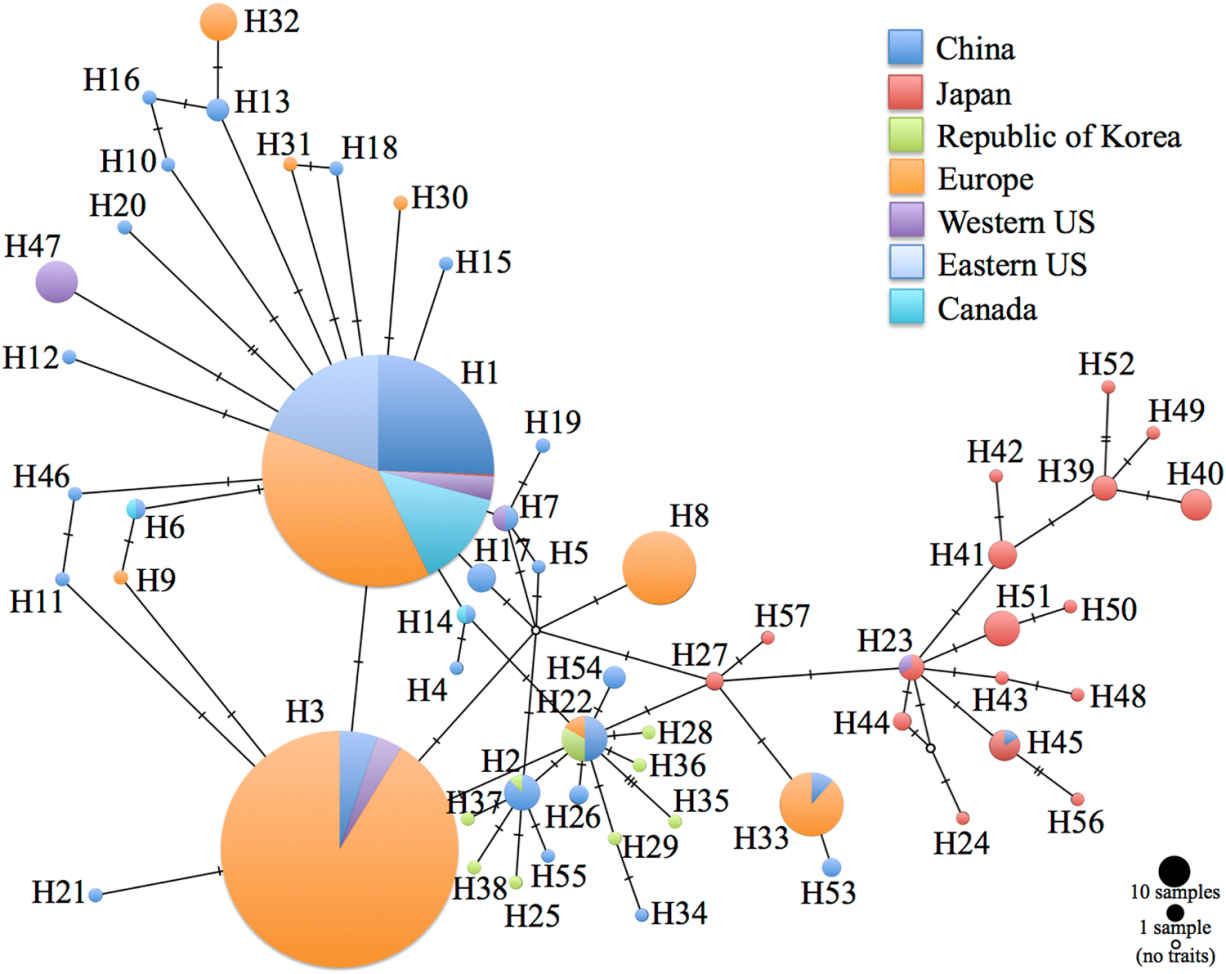
A CO1 haplotype network generated for BMSB, with geographic representation for each haplotype.

The analysis using CO1 sequences from specimens used in Xu *et al*.^31^ supported their conclusion that China was the source population for the initial introduction of BMSB into the US (probability (p) = 0.89) (Table 2). When we tested our first question, which was whether there was more than one introduction event into North America (Table 3), we found that Canadian populations were likely also sourced from China, with a probability of 0.76, instead of Japan or the Republic of Korea (Table 2). The haplotype network further supported this finding, with the Canada haplotypes found only within the China cluster of the network (Fig. 1). When we tested scenarios with admixture to see if it was more probable that the US, or a mix of the US and China, colonized Canada rather than China alone we found that the US only colonization scenario was the only unlikely scenario of the four, with the other three scenarios very similar in probability (Table 2).

**Table 2:** Probability and 95% credible interval for all Approximate Bayesian Computation scenarios used throughout the study, along with confidence in scenario choice.

Experiment	Prob.	95% CI	Conf.
Pre-2008 NE US source determination			0.869
1: China source	0.8910	[0.7925, 0.9894]	
2: Japan source	0.0625	[0.0000, 0.1429]	
3: Korea source	0.0466	[0.0000, 0.1018]	
Canada source determination			0.796
1: China source	0.7561	[0.6415, 0.8708]	
2: Japan source	0.1150	[0.0373, 0.1926]	
3: Korea source	0.1289	[0.0452, 0.2126]	
Canada source w/admixture			0.551
1: China only source	0.3087	[0.2592, 0.3583]	
2: US only source	0.1284	[0.0934, 0.1634]	
3: China + East US	0.3103	[0.2677, 0.3528]	
4: China + Northwest US	0.2526	[0.2078, 0.2974]	
Northwestern US source determination			0.776
1: China source	0.8841	[0.8292, 0.9389]	
2: Japan source	0.0364	[0.0133, 0.0596]	
3: Korea source	0.0795	[0.0347, 0.1243]	
Northwestern US source w/admixture			0.509
1: China source	0.5538	[0.4574, 0.6502]	
2: China + Japan source	0.1262	[0.0697, 0.1828]	
3: China + Korea source	0.3200	[0.2261, 0.4138]	
Introduction to California			0.352
1: Separate introduction from China	0.0764	[0.0000, 0.1874]	
2: Dispersal from Northwestern US	0.0890	[0.0308, 0.1471]	
3: Dispersal from Eastern US	0.1071	[0.0484, 0.1657]	
4: Introduced to California then spread to Eastern US	0.0891	[0.0299, 0.1484]	
5: Mixture of Northwestern and East US	0.1345	[0.0758, 0.1933]	
6: Mixture of separate China introduction and Eastern US	0.1988	[0.1267, 0.2709]	
7: Mixture of separate China introduction and Northwestern US	0.3051	[0.2180, 0.3922]	
↓			
3: Dispersal from Eastern US	0.1258	[0.0726, 0.1791]	
5: Mixture of Northwestern and East US	0.1894	[0.1217, 0.2570]	
6: Mixture of separate China introduction and Eastern US	0.2433	[0.1537, 0.3329]	
7: Mixture of separate China introduction and Northwestern US	0.4415	[0.3345, 0.5485]	
Europe source determination (minus Greece)			0.746
1: China source	0.9570	[0.9326, 0.9814]	
2: Japan source	0.0179	[0.0047, 0.0312]	
3: Korea source	0.0251	[0.0077, 0.0426]	
Likelihood of bridgehead from US to Europe			0.758
1: China source	0.7742	[0.6404, 0.9081]	
2: Bridgehead from Eastern US	0.0980	[0.0000, 0.5300]	
3: Bridgehead from Western US	0.1278	[0.0418, 0.2137]	
Greece source determination			0.762
1: China source	0.5948	[0.5170, 0.6726]	
2: Japan source	0.0332	[0.0000, 0.1435]	
3: Korea source	0.3720	[0.2544, 0.4896]	
Bridgehead from US to Emilia-Romagna, Italy			0.779
1: Dispersal from Europe	0.0731	[0.0222, 0.1240]	
2: Bridgehead from US	0.7078	[0.6119, 0.8037]	
3: Separate introduction from China	0.2191	[0.1310, 0.3071]	
Introduction history of Greece and Hungary			0.530
1: Greece from China only	0.2265	[0.1813, 0.2716]	
2: Mixture from Hungary and China	0.2539	[0.2060, 0.3019]	
3: China source that spread to Hungary	0.5196	[0.4583, 0.5809]	

A downward arrow indicates a subsequent analysis with scenarios with probabilities below 0.1 excluded.

**Table 3:** The four principal questions being asked throughout this study.

1	Were there multiple introductions into North America, and if so what were the sources?
2	What was the source population for the initial introduction (i.e. through Switzerland) to Europe?
3	Was there a bridgehead event from the United States to Europe?
4	Were there multiple introductions into Europe, and if so what were the sources?

These questions shape the smaller questions that become the scenarios we model throughout the paper.

**Figure 2:**
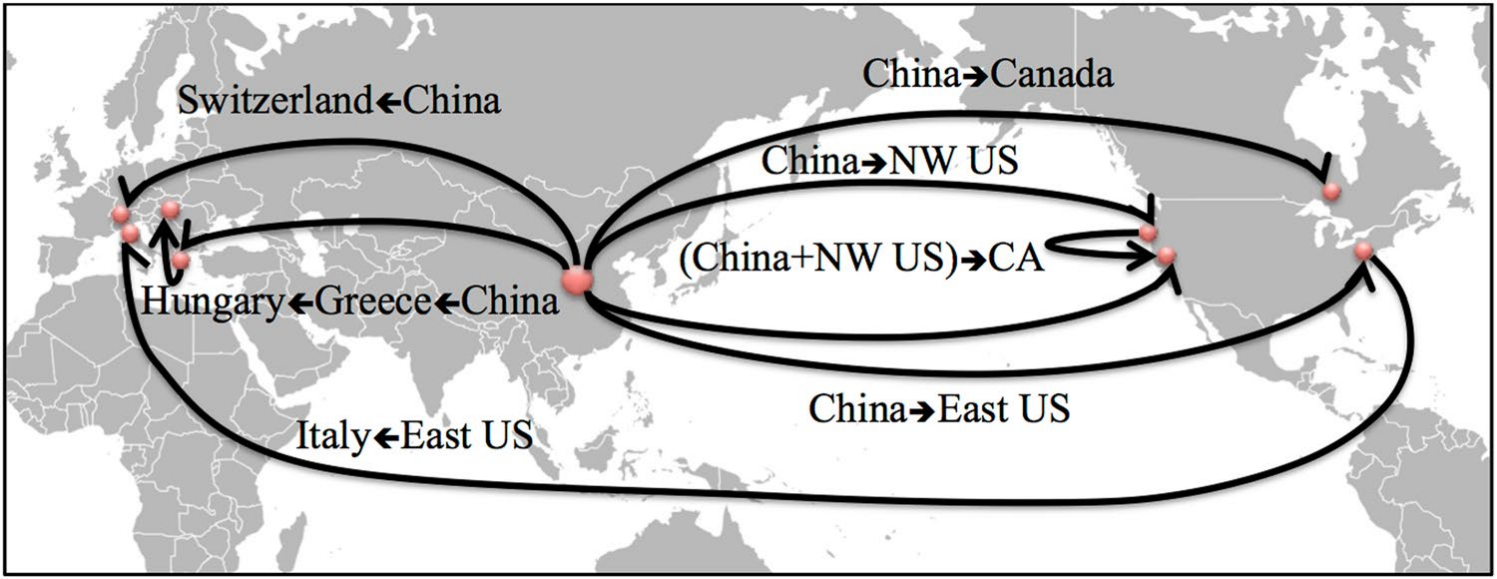
Map of the most likely BMSB invasion pathways connecting native and established populations across the globe, summarizing the results of our ABC analyses. Red dots on the map indicate the relevant native or established populations that are part of separate invasion pathways. The map’s pathways are directional and labeled with the source population(s) listed, along with the calculated probabilities. The abbreviation NW refers to the northwestern population within the United States (US), CA refers to the US state of California, and the small arrow within the text provides additional clarity regarding the direction of the pathway. The base map, titled Blank Map Pacific World, was created by Dmthoth (https://commons.wikimedia.org/wiki/File:Blank_Map_Pacific_World.svg) and altered to show the invasion pathways.

**Figure 3:**
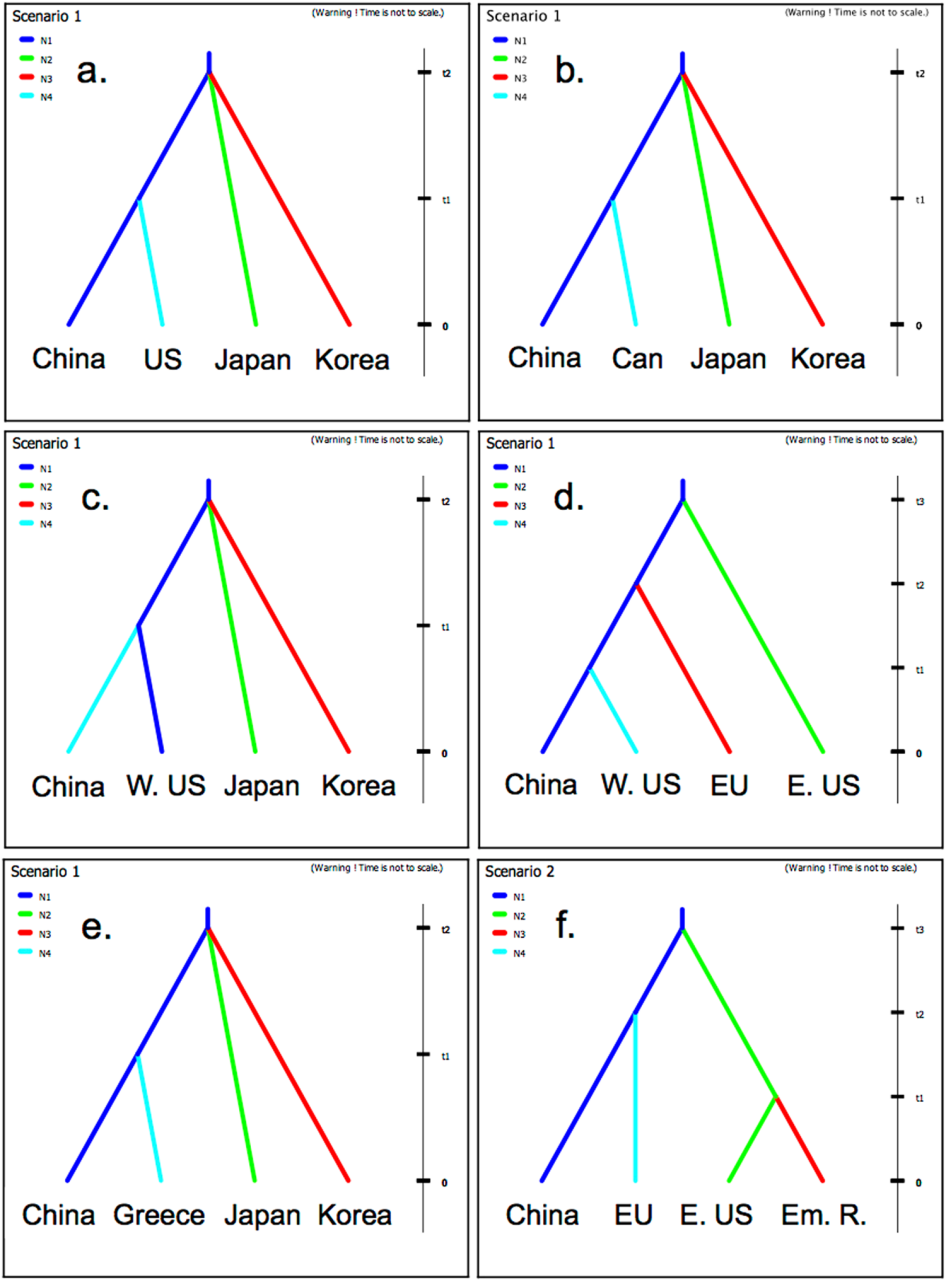
Approximate Bayesian Computation scenario outputs for the six significant scenarios with confidence values above 0.75: (**a**) native source population for the pre-2008 US dataset; (**b**) native source population for Canada; (**c**) native source population for the Western US; (**d**) introduction scenario for EU; (**e**) native source population for Greece; (**f**) bridgehead from US to Emilia-Romagna, Italy. E. US and W. US represent Eastern and Western United States, respectively. Can is an abbreviation for Canada. Em. R. is an abbreviation for Emilia-Romagna, Italy. All scenarios shown here are ordered based on experiment order in Table 2.

Tests for multiple introductions to the US indicated that Northwestern US populations (in Washington and Oregon) were also likely separate introductions from China (p = 0.88) (Fig. 2, Fig. 3, Table 2). We observed no overlap of haplotypes between the United States and Korea and a single haplotype overlap (H23) between the United States and Japan within the observed haplotype network (Fig. 1), and found a low probability that this population was a result of admixture from these different native populations via ABC analysis (Table 2). Given the disparity in haplotype make-up and diversity between the western states (5 haplotypes) and eastern states (1 haplotype, Table 2), we decided it was unnecessary to conduct a formal test to see if the western populations of BMSB were the result of a dispersal event from the east. Instead, we focused on the seven possible scenarios for the introduction to California (Table 2). Of these, the scenario of California being a mix of the northwestern states and China (question 1 g in Methods) was significantly different from all except the scenario of California being a mix of the eastern states and China (question 1 f), with the latter not being significantly different from the remaining five (Table 2). When we re-ran the analysis after excluding all scenarios below a 0.10 probability (e.g. scenarios for questions 1a, 1b, and 1d), we found the scenario for question 1 g to be significantly more likely than the rest (Table 2, Fig. 2). However, confidence for this result was below 0.4 (Table 2).

Regarding questions two and three, which were to identify the source(s) of the introduction into Europe (excluding Greece) and to assess the likelihood of a bridgehead from the US to Europe (Table 3), our results indicated that the European populations of BMSB examined were sourced from China with a high probability of 0.96 (Table 2, Fig. 3). When we tested for the possibility of a bridgehead from either the US’ eastern or western populations into Europe we found there was little support for this (p = 0.10 and p = 0.13 respectively) and that China was again the likely source, with a 0.77 probability (Table 2, Fig. 2). China was also the likely source of the Greece population with a probability of 0.60 (Table 2, Fig. 2, Fig. 3), which was significantly different from a source in Korea (p = 0.37) or Japan (p = 0.03).

Next, we addressed question four, which was to determine whether there were multiple introductions into Europe and identify the sources (Table 3). We did so by first testing the hypothesis that the Italian Emilia-Romagna population was the result of a bridgehead from the US (p = 0.71) (Fig. 2) rather than natural dispersal from Northern Italy or a separate introduction from China (p = 0.07 and p = 0.22, respectively) (Table 2, Fig. 3). Second, we tested the possibilities of Greece populations being a separate introduction from only China, a mix from China and nearby Hungary, and a separate introduction from China to Greece that subsequently became the source of BMSB to Hungary. We found that Greece was likely sourced from China and then became the source of the introduction to Hungary (p = 0.52) (Table 2). This scenario was significantly more likely than an introduction from both China and Hungary to Greece (p = 0.25) or from only China to Greece without spreading to Hungary (p = 0.23), with a confidence just over 0.5 (Table 2).

## Discussion

We developed an analysis that included representative infestations of non-native brown marmorated stink bugs (BMSB – *Halyomorpha halys*) from across the world, as well as multiple populations from the native range, to assess the most probable worldwide invasion pathways. To accomplish this, we combined published genetic data from four studies of BMSB in non-native regions using the CO1 marker^31, 38–40^, added new sequences from both the introduced and native range as needed and feasible, and performed a Templeton, Crandall, and Sing (TCS) haplotype network analysis^41^ and Approximate Bayesian Computation (ABC) analyses to quantify pathway probabilities. Sadly, our analyses excluded published sequences that did not match the CO1 barcoding region (e.g. Zhu *et al*.^42^) as well as specimens from the most recent discovered populations in Serbia, Romania, Russia, ARA, Georgia, and Bulgaria due to lack of access to specimens of BMSB from those populations. Though ABC does have its drawbacks (i.e., computational power, potential user scenario bias, and some limitations on the questions that can be effectively asked), we carefully tried to minimize bias in our study by modeling all scenarios under numerous alternative hypotheses to prevent forcing a desired outcome. Furthermore, we kept many questions at the country level due to low sample size in several parts of the native range and lack of genetic differentiation at the barcode CO1 locus. Case in point, unlike Xu *et al*.^31^ we did not attempt to identify the specific location(s) of origin within China. Instead, the worldwide analyses provide insights into broader patterns of expansion of this economically important pest species.

Although there was a stark contrast in the genetic makeup of eastern and Western US populations (Table 1), this alone was not conclusive evidence of multiple introductions since the eastern population could have been a result of dispersal from the western states. We found this possibility unlikely, and our results instead indicate that the Eastern and Western US experienced separate introduction events of BMSB from China. In addition, we also found that populations in California appear to be a mix from both China and populations in the northwestern states (Washington and Oregon), indicating that at least three separate introduction events from China may have occurred to the US. However, likely due to the lack of variation at the CO1 locus, confidence in the specific scenarios underlying the expansion into California was relatively low (Table 2) and warrants further exploration with additional and more variable loci (e.g. microsatellites) and/or more extensive sequencing of nuclear regions.

We also found that Canada was likely sourced from a separate introduction from China. However, our analyses could not provide a single clear scenario for the complete introduction history of BMSB into Canada, since the China only scenario was not significantly different from the China and US mixed population scenarios (Table 2). Given the proximity of Canada’s invaded range to the northern range found in the US, it is not completely surprising that this could be the case.

In contrast, our results clearly indicated that China was the source of the introduction into Switzerland that subsequently spread to neighboring European countries. We also found support for a bridgehead event from the Eastern US into Emilia-Romagna, Italy, reinforcing the hypothesis made by Cesari *et al*.^40^ regarding the occurrence of multiple introductions into Italy. This is a particularly interesting finding given the documented extensive economic injury to tree fruits and nuts in locations with high proportions of the H1 haplotype in the US and in Emilia-Romagna, Italy^22, 43^. A high frequency of the H1 haplotype may be indicative of a phenotype either more prone to become invasive or adapted to tree fruits as a primary food resource - hypotheses that warrant further behavioral, physiological, and genomic analyses.

Greece was analyzed separately from the remainder of Europe due to its observed higher level of genetic diversity. Again, we found significant evidence of a direct introduction from China to Greece that excluded other European populations as sources. Additionally, we found that the population in Hungary was most likely due to a dispersal event from Greece rather than a continued dispersal event from Western Europe. This indicates at least three separate introduction events from China into Europe, two in Western Europe and one in Eastern Europe.

Although we answered the four primary questions (Table 3) our analyses are based on a single maternally inherited locus, CO1, which may underrepresent existing genetic variation. The addition of nuclear data, such as microsatellites or a NextGen based array of Single Nucleotide Polymorphisms (SNPs), should provide much needed information from both sides of the parental lineage that can be paired with the CO1 data for more in depth analyses.

While we recommend evaluating the invasion pathways of the more recent detections into other parts of Europe (i.e. Serbia, Romania, Russia, ARA, Georgia, and Bulgaria), and found evidence of one long-distance bridgehead event, most critically our analyses indicate that the expansion of BMSB across the world is still primarily sourced from China (Fig. 2 **)**. This is an important result because numerous invasion events all from China, possibly even from the same location, in a relatively short amount of time, indicate the existence of an export pathway that if identified could be closed leading to major decreases in the spread of BMSB across the World. The next step then is to identify the invasion vectors, and while careful vetting of cargo potentially harboring BMSB may be onerous, it may prevent the much higher economic losses associated with this agricultural pest continuing to spread globally.

## Methods

### Global dataset assembly

To direct our approach and ascertain which loci had been used, we first reviewed the literature and downloaded sequence data from Genbank for the four genetic analyses of the expansion of BMSB in North America and Europe that contained both native and non-native BMSB populations^31, 38–40^. The study performed in the US primarily sequenced regions of the cytochrome oxidase 2 (CO2) locus and the control region (CR), with very limited sequencing in the cytochrome oxidase 1 (CO1)^31^. In contrast, Canadian and European studies examined sections of CO1, CO2, and Cytochrome b (Cyt b)^38–40^, though CO1 was the only common locus between these studies (Table 4). Since the most often sequenced locus was CO1 we decided to focus on it for the analysis.

**Table 4:** Mitochondrial DNA loci used in population genetic studies of brown marmorated stink bug by country, with the relevant authors conducting them on the right.

Country	Loci				References
(no. of specimens)	CO1	CO2	CR	Cty - b	
China	X	X	X	X	Xu *et al*.^31^, Gariepy *et al*.^39^
Japan	X*	X	X	X*	Xu *et al*.^31^, Gariepy *et al*.^39^
Republic of Korea	X*	X	X		Xu *et al*.^31^, Gariepy *et al*.^39^
United States	X*	X	X		Xu *et al*.^31^
Canada	X			X	Gariepy *et al*.^39^
Switzerland	X	X		X	Gariepy *et al*.^39^, Cesari *et al*.^40^
Italy	X	X			Cesari *et al*.^40^
France	X				Gariepy *et al*.^38^
Hungary	X				Gariepy *et al*.^38^
Greece	X				Gariepy *et al*.^38^

*Designates loci with five or few samples sequenced.

We started by sequencing CO1 for many of the specimens from the US and Asia in Xu *et al*.^31^, but since the most recent US samples in the dataset were from 2008, new specimens from across the US range were added (Table 1). Additionally, we obtained samples from two new populations in Japan and one new population from the Republic of Korea in order to have a better representative sample of the native range. Newly acquired samples were received dry (with a desiccant present) or in 95–100% molecular grade ethanol. All dry specimens were stored at −20 °C while those in ethanol were stored at room temperature. To extract the genomic DNA (gDNA) we used flame sterilized tweezers to pull one leg with the underlying thoracic muscle tissue connected from each specimen^31^. We then extracted total gDNA with a DNeasy blood and tissue kit using the provided protocol (Qiagen Sciences, Germantown, MD, USA), with Proteinase K incubation overnight (minimum of 8 hours) to ensure complete digestion.

We amplified the 685 bp of the CO1 standard barcode region^44^ used by the European studies^38–40^ with primers LCO1490 (5′-GGTCAACAAATCATAAAGATATTGG-3′) and HCO2198 (5′-TAAACTTCAGGGTGACCAAAAAATCA-3′). Amplifications were accomplished in 20 μl reactions consisting of 1 × PCR buffer (10 mM Tris-HCl, pH 8.3, and 50 mM KCl), 2.5 mM MgCl_2_, 100 μM of each dNTP, 200 nM of each primer, 1 unit of Amplitaq Gold DNA polymerase (Applied Biosystems, Life Technologies, Carlsbad, CA) and approximately 20 ng gDNA. The protocol was optimized to run at an initial denaturing temperature of 96 °C for 10 minutes, followed by 45 cycles of the following steps: denaturing at 96 °C for 20 seconds, annealing at 50 °C for 30 seconds, and extension at 72 °C for 30 seconds. Final extension was completed at 72 °C for 2 minutes. All PCRs were run on a Veriti 96-Well Thermal Cycler (Applied Biosystems, Life Technologies, Carlsbad, CA). We visualized amplifications in a 1% agarose gel with Ethidium Bromide, and selected DNA fragments of appropriate size for sequencing. Successful amplicons were cleaned using ExoSAP-IT (Affymetrix, OH), and mixes of 25pmoles of primer and 20 ng of template DNA were sent for cycle sequencing and sizing (Genscript, Piscataway, NJ, USA). Cycle sequencing was performed using both the forward and reverse primers to create a consensus sequence and increase haplotype reliability. Chromatograms were cleaned and aligned in Sequencer 5.1 (GeneCodes, Ann Arbor, MI). All sequences were evaluated for insertions and deletions, as well as translated to amino acids to check for stop codons and limit the presence of nuclear copies^45^. We then exported the full CO1 contig in nexus format and created a TCS haplotype network using the program PopArt^46^ (Fig. 1).

### ABC performance evaluation

Once we obtained a global dataset of variants of CO1 in BMSB we conducted several analyses using Approximate Bayesian Computation (ABC). Specifically, through our analyses we sought to address four major questions regarding the spread of BMSB globally (Table 3 **)**. These questions were chosen not only to check assumptions made in the literature regarding the invaded range in prior studies^31, 38–40^, but also to address hypotheses only testable with a worldwide dataset. We modeled these invasion pathway scenarios based on the initial findings of the genetic data, possible pathways hypothesized in other papers^31, 38–40^, and other *a priori* factors that can affect introductions (e.g. proximity and natural dispersal, bridgehead effects, etc.). We then compared appropriate scenarios against each other to quantitatively determine which had the highest likelihood of having taken place based on logistic posterior probabilities.

We selected the program DIYABC to carry out our analyses^19, 20^. Before addressing our four major questions, we sought to evaluate the performance of this analysis method, based on a single mitochondrial locus, by testing the hypothesis that China was the native source population of the US introduction, as proposed by Xu *et al*.^31^ using two loci. For parity, we limited the analysis to the same specimens analyzed at the time. To accomplish this, we developed three simple scenarios and tested them against each other: (1) US haplotypes were sourced from the China population; (2) US haplotypes were sourced from the Japan population; and (3) US haplotypes were sourced from the Republic of Korea population. All three scenarios were weighted equally, with priors set to fit a uniform distribution and the US effective population size restricted to be lower than the native populations. We determined the evolutionary model to be used via the program PartitionFinder 1.1.1^47^ in Python v2.7 under an unpartitioned whole gene model scheme with a MrBayes filter. We did so to ensure the evolutionary models selected by PartitionFinder would be limited to the model options supported by the DIYABC software. Once complete, we used the recommended Hasegawa, Kishino and Yano (HKY) model^48^ and set priors as shown in Table 5. We set population parameters to uniform, with default settings, set the condition for the US population (N4) to be less than all other native populations (e.g. N1, N2, and N3) and ran the program for 1 million iterations.

**Table 5:** Prior distributions used for all ABC analyses.

Description	Prior distribution			
Mutation Parameters				
Mutation model	HKY	10% invariant sites		Shape (2)
Mean mutation rate	Uniform	(min) 1.00E-7	(max) 1.00E-5	
Indiv. locus mutation rate	Gamma	(min) 1.00E-7	(max) 1.00E-5	Shape (2)
Mean coefficient (k C/T)	Uniform	(min) 1.5	(max) 20	
Indiv. locus coefficient (k C/T)	Gamma	(min) 1.5	(max) 20	Shape (2)

Mutation parameters refer to selected DNA mutation model, distributions used, and bounds for said distributions within the model validation screen.

Once the computations were complete, we performed a pre-evaluation of the scenarios and prior combinations using a principal component analysis (PCA) approach within the program. We inspected both the PCA plot and the numerical values and proceeded only when the observed dataset was within the cloud of pseudo-observed datasets (PODs) and the numerical values displayed summary statistics for simulated data with very few values below the observed dataset (indicated by having very few or no three-star – i.e. highly significant - exceptions across most or all scenarios). We then computed the posterior probabilities for each of the scenarios to assess which was most likely to have occurred given our dataset, and evaluated confidence in scenario choice using posterior based error.

### Testing invasion pathway hypotheses with ABC

After evaluating the performance of this method by comparing our initial findings against previously proposed results^31^, we began developing models for our four questions regarding the global invasion of BMSB. To address question one (Table 3) we first examined possible native sources for the population in Canada, then tested to see if it was more likely to have come from the US, a native source, or a mix of the two (Figure S1a). Next, we assessed if the haplotype identity and diversity found in the western portion of the US could have been attributed to natural dispersal from the eastern portion, or if another introduction from the native range was more likely. To do so we first determined the likely native source(s) of the western populations, then tested if that native source had a higher probability of having occurred than a dispersal event from the eastern half, or if it was a combination of the two (Figure S1b). However, it is possible that the reverse is true, and the eastern population was the result of a dispersal event from California. Therefore, after determining the likely native source of the western states we split them into two groups, consisting of the northwestern states (Washington and Oregon) and California, and created seven scenarios (Table 2, Figure S1c): 1) California was a separate introduction sourced from China; 2) California was sourced from a separate introduction event to the northwestern states; 3) California was sourced from the eastern states; 4) California was the site of the initial introduction site of BMSB to the US and then spread to the eastern states; 5) California is a mix of Northwest and Eastern US populations; 6) California is a mix of an introduction from China and a dispersal event from the Eastern US; and 7) California is a mix of a separate introduction event from China and a dispersal event from the Northwestern US. The above experiments had their priors set identical to our first US experiment (Table 5), with the non-native populations (US and Canada) effective population again restricted to being lower than all native populations, run for 1 million iterations, and scenario confidence calculated using posterior based error.

We addressed both questions two and three (Table 3) by first developing three simple scenarios, each testing the probability of a native population being the source of the introduction into Europe (excluding Greece), to see what the most likely native source could have been, again following the same prior parameters throughout (Table 5). Once the likely native source was determined we tested the possibility of a bridgehead from the US to the early introduction of Europe by comparing the most likely modeled scenarios against the native source scenario (Figure S1d).

We addressed question four (Table 3) by first testing a hypothesis from Cesari *et al*.^40^ that the southern-most population in Italy (Emilia-Romagna) was a bridgehead event from the US, while the northern most population (Lombardi) was a dispersal from broader Europe. We tested this with two scenarios, one involving a dispersal event from Northern Italy to Southern Italy and the other a bridgehead event from the Eastern US into Southern Italy (Figure S1e). Lastly, due to the very different haplotype identity and diversity detected in Greece, we tested multiple scenarios for its colonization, first by determining the most likely native source(s). Once we determined the native source(s) we created four scenarios to evaluate: 4a) the possibility of the Greek population having been a separate introduction from the native range; 4b) a dispersal event from its closest European country with an established population (Hungary); 4c) a mixture of scenarios 4a and 4b; or 4d) a separate introduction from the native range to Greece that then spread to Hungary (Figure S1f).

### Data accessibility

Accession numbers to all downloaded and generated sequence data analyzed are provided within a supplementary table (Table S1), sorted by haplotype. Sequence accession numbers are for submissions to Genbank.

## Electronic supplementary material

Supplementary Table and Figure

## References

[1] Blackburn TM (2011). A proposed unified framework for biological invasions. Trends in Ecology & Evolution.

[2] Roman J, Darling JA (2007). Paradox lost: genetic diversity and the success of aquatic invasions. Trends in Ecology & Evolution.

[3] Lockwood, J. L., Hoopes, M. F. & Marchetti, M. P. *Invasion ecology*. (John Wiley & Sons, 2013).

[4] Pimentel D, Zuniga R, Morrison D (2005). Update on the environmental and economic costs associated with alien-invasive species in the United States. Ecological economics.

[5] Hänfling B, Carvalho GR, Brandl R (2002). mt-DNA sequences and possible invasion pathways of the Chinese mitten crab. Marine Ecology Progress Series.

[6] Brown JE, Stepien CA (2009). Invasion genetics of the Eurasian round goby in North America: tracing sources and spread patterns. Molecular Ecology.

[7] Ghabooli S (2013). Invasion Pathway of the Ctenophore *Mnemiopsis leidyi* in the Mediterranean Sea. PLoS ONE.

[8] Kaufman MG, Fonseca DM (2014). Invasion biology of *Aedes japonicus japonicus* (Diptera: Culicidae). Annual review of entomology.

[9] Ascunce MS (2011). Global invasion history of the fire ant *Solenopsis invicta*. Science.

[10] Leung B (2002). An ounce of prevention or a pound of cure: bioeconomic risk analysis of invasive species. Proceedings of the Royal Society of London B: Biological Sciences.

[11] Valentin, R. E., Maslo, B., Lockwood, J. L., Pote, J. & Fonseca, D. M. Real‐time PCR assay to detect brown marmorated stink bug, *Halyomorpha halys* (Stål), in environmental DNA (eDNA). *Pest management science* (2015). 10.1002/ps.4217 26732613

[12] Simberloff D (2013). Impacts of biological invasions: what’s what and the way forward. Trends in Ecology & Evolution.

[13] Estoup A, Guillemaud T (2010). Reconstructing routes of invasion using genetic data: why, how and so what?. Molecular Ecology.

[14] Lombaert E (2010). Bridgehead Effect in the Worldwide Invasion of the Biocontrol Harlequin Ladybird. PLoS ONE.

[15] Navarro J-C, Quintero L, Zorrilla A, González R (2013). Molecular tracing with mitochondrial ND5 of the invasive mosquito *Aedes* (Stegomyia) *albopictus* (Skuse) in Northern South America. J Entomol Zool Stud.

[16] Darling JA, Bagley MJ, Roman J, Tepolt CK, Geller JB (2008). Genetic patterns across multiple introductions of the globally invasive crab genus *Carcinus*. Molecular Ecology.

[17] Costa-da-Silva ALd, Capurro ML, Bracco JE (2005). Genetic lineages in the yellow fever mosquito *Aedes* (Stegomyia) *aegypti* (Diptera: Culicidae) from Peru. Memórias do Instituto Oswaldo Cruz.

[18] Mousson L (2005). Phylogeography of *Aedes* (Stegomyia) *aegypti* (L.) and *Aedes* (Stegomyia) *albopictus* (Skuse)(Diptera: Culicidae) based on mitochondrial DNA variations. Genetical research.

[19] Cornuet J-M (2008). Inferring population history with DIY ABC: a user-friendly approach to approximate Bayesian computation. Bioinformatics.

[20] Cornuet J-M (2014). DIYABC v2. 0: a software to make approximate Bayesian computation inferences about population history using single nucleotide polymorphism, DNA sequence and microsatellite data. Bioinformatics.

[21] Hoebeke, E. R. & Carter, M. E. *Halyomorpha halys* (Stal)(Heteroptera: Pentatomidae): A polyphagous plant pest from asia newly detected in North America. *Proc. Entomol. Soc. Wash* (2003).

[22] Rice KB (2014). Biology, Ecology, and Management of Brown Marmorated Stink Bug (Hemiptera: Pentatomidae). Journal of Integrated Pest Management.

[23] Milonas P, Partsinevelos G (2014). First report of brown marmorated stink bug *Halyomorpha halys* Stål (Hemiptera: Pentatomidae) in Greece. EPPO Bulletin.

[24] Vetek G, Papp V, Haltrich A, Redei D (2014). First record of the brown marmorated stink bug, *Halyomorpha halys* (Hemiptera: Heteroptera: Pentatomidae), in Hungary, with description of the genitalia of both sexes. Zootaxa.

[25] Leskey TC (2012). Pest status of the brown marmorated stink bug, *Halyomorpha halys* in the USA. Outlooks on Pest Management.

[26] Nielsen AL, Hamilton GC (2009). Seasonal occurrence and impact of *Halyomorpha halys* (Hemiptera: Pentatomidae) in tree fruit. Journal of Economic Entomology.

[27] Leskey, T. C., Short, B. D., Butler, B. R. & Wright, S. E. Impact of the invasive brown marmorated stink bug, *Halyomorpha halys* (Stål), in mid-Atlantic tree fruit orchards in the United States: case studies of commercial management. *Psyche: A**Journal of Entomology* **2012** (2012).

[28] Polk, D. F. The Impact of brown marmorated stink bug in NJ tree fruit 2010 In *Proc. 86th Cumberland-Shenandoah Fruit Workers Conference* Winchester, VA November (2011).

[29] Hedstrom C, Shearer P, Miller J, Walton V (2014). The effects of kernel feeding by *Halyomorpha halys* (Hemiptera: Pentatomidae) on commercial hazelnuts. Journal of economic entomology.

[30] Wiman, N. G., Parker, J. E., Rodriguez-Saona, C. & Walton, V. M. Characterizing damage of brown marmorated stink bug (Hemiptera: Pentatomidae) in blueberries. *Journal of economic entomology*, tov036 (2015). 10.1093/jee/tov036 26470241

[31] Xu J, Fonseca DM, Hamilton GC, Hoelmer KA, Nielsen AL (2014). Tracing the origin of US brown marmorated stink bugs. Halyomorpha halys. Biological invasions.

[32] Macavei LI (2015). First detection of *Halyomorpha halys* Stål, a new invasive species with a high potential of damage on agricultural crops in Romania. *Lucrări Ştiinţifice, Universitatea de Ştiinţe Agricole şi Medicină Veterinară” Ion Ionescu de la Brad” Iaşi, Seria*. Agronomie.

[33] Šeat J (2015). *Halyomorpha halys* (Stål, 1855)(Heteroptera: Pentatomidae) a new invasive species in Serbia. Acta entomologica serbica.

[34] Gapon D (2016). First records of the brown marmorated stink bug *Halyomorpha halys* (Stål, 1855)(Heteroptera, Pentatomidae) in Russia, Abkhazia, and Georgia. Entomological Review.

[35] Simov N (2016). The invasive brown marmorated stink bug *Halyomorpha halys* (Stål, 1855)(Heteroptera: Pentatomidae) already in Bulgaria. Ecologica Montenegrina.

[36] Wermelinger B, Wyniger D, Forster B (2008). First records of an invasive bug in Europe: *Halyomorpha halys* Stål (Heteroptera: Pentatomidae), a new pest on woody ornamentals and fruit trees?. Mitteilungen der Schweizerischen entomologischen Gesellschaft.

[37] Arnold, K. *Halyomorpha halys* (Stal, 1855), a stink bug species newly detected among the European fauna (Insecta: Heteroptera, Pentatomidae, Pentatominae, Cappaeini). *Mitteilungen des Thuringer Entomologenverbandes* **16** (2009).

[38] Gariepy, T., Bruin, A., Haye, T., Milonas, P. & Vétek, G. Occurrence and genetic diversity of new populations of Halyomorpha halys in Europe. *Journal of Pest Science*, 1–10 (2015).

[39] Gariepy T, Haye T, Fraser H, Zhang J (2014). Occurrence, genetic diversity, and potential pathways of entry of *Halyomorpha halys* in newly invaded areas of Canada and Switzerland. Journal of pest science.

[40] Cesari M (2015). A pest alien invasion in progress: potential pathways of origin of the brown marmorated stink bug *Halyomorpha halys* populations in Italy. Journal of Pest Science.

[41] Templeton AR, Crandall KA, Sing CF (1992). A cladistic analysis of phenotypic associations with haplotypes inferred from restriction endonuclease mapping and DNA sequence data. III. Cladogram estimation. Genetics.

[42] Zhu, G.-P. *et al*. Range wide molecular data and niche modeling revealed the Pleistocene history of a global invader (*Halyomorpha halys*). *Scientific reports* **6** (2016). 10.1038/srep23192 PMC4800403 26996353

[43] Bariselli M, Bugiani R, Maistrello L (2016). Distribution and damage caused by *Halyomorpha halys* in Italy. EPPO Bulletin.

[44] Ekrem T, Willassen E, Stur E (2007). A comprehensive DNA sequence library is essential for identification with DNA barcodes. Molecular phylogenetics and evolution.

[45] Egizi A, Healy SP, Fonseca DM (2013). Rapid blood meal scoring in anthropophilic *Aedes albopictus* and application of PCR blocking to avoid pseudogenes. Infection, Genetics and Evolution.

[46] Leigh JW, Bryant D (2015). popart: full-feature software for haplotype network construction. Methods in Ecology and Evolution.

[47] Lanfear R, Calcott B, Ho SY, Guindon S (2012). PartitionFinder: combined selection of partitioning schemes and substitution models for phylogenetic analyses. Molecular biology and evolution.

[48] Hasegawa M, Kishino H (1985). & Yano, T.-a. Dating of the human-ape splitting by a molecular clock of mitochondrial DNA. Journal of Molecular Evolution.

