# Supplementary material for: Global invasion network of the brown marmorated stink bug, Halyomorpha halys
Source: Sci Rep. 2017 Aug 29;7:9866. doi: 10.1038/s41598-017-10315-z (PMC5575200; doi:10.1038/s41598-017-10315-z)

Global invasion network of the brown marmorated stink bug, *Halyomorpha halys*

Rafael Valentin[1,5], Anne L. Nielsen[2], Nik G. Wiman[3], Doo-Hyung Lee[4], Dina M. Fonseca[1,2]

Author affiliations:
[1]Department of Ecology, Evolution & Natural Resources, Rutgers University, 14 College Farm Rd., New Brunswick, NJ 08901, USA

[2]Department of Entomology, Rutgers University, 93 Lipman Drive, New Brunswick, NJ 08901, USA

[3]Department of Horticulture, Oregon State University, 4017 ALS Building, Corvallis, OR 97330, USA

[4]Department of Life Sciences, Gachon University, Seongnam-si, Gyeonggi-do, South Korea

[5]**Corresponding author**: Rafael Valentin, Ecology and Evolution Graduate Program, 14 College Farm Rd., New Brunswick, NJ 08901, USA; Rafael.Valentin@Rutgers.edu

Table S1. BMSB CO1 haplotype table sorted by country, with total number of samples from each country below the name, and accession numbers to the Genbank sequences provided. Samples corresponding to specific haplotypes in bold represent new haplotypes found in the US post-2008 analysis.

	China (158)	Japan (44)	Korea (12)	East US (70)	West US (38)	Canada (51)	Switzerland (223)	Italy (40)	France (139)	Hungary (84)	Greece (57)	Accession number
H1	92	1		70	12	49	2	32	1	83	18	KF273380.1
H2	7		1									KF273381.1
H3	19				**13**		191	7	136	1	4	KF273382.2
H4	1											KF273383.2
H5	1											KF273384.1
H6	1					1						KF273385.1
H7	1				1							KF273386.2
H8							31	1	2			KF273387.1
H9							1					KF273388.1
H10	1											KF273389.1
H11	1											KF273390.1
H12	1											KF273391.1
H13	3											KF273392.1
H14	1					1						KF273393.1
H15	1											KF273394.1
H16	1											KF273395.1
H17	5											KF273396.1
H18	1											KF273397.1
H19	1											KF273398.1
H20	1											KF273399.1
H21	1											KF273400.1
H22	6		4								2	KF273401.1
H23		2			**1**							KF273402.1
H24		1										MF537219

H27		2				MF537222
H28			1			MF537223
H29			1			MF537224
H30					1	KR070749.1
H31					1	KR070748.1
H32					8	KR070750.1
H33	3				23	KR070751.1
H34	1					MF537225
H35			1			MF537226
H36			1			MF537227
H37			1			MF537228
H38			1			MF537229
H39		4				MF537230
H40		6				MF537231
H41		5				MF537232
H42		1				MF537233
H43		1				MF537234
H44		2				MF537235
H45	1	5				MF537236
H46	1					MF537237
H47				**11**		MF537238
H48		1				MF537239
H49		1				MF537240
H50		1				MF537241
H51		8				MF537242
H52		1				MF537243
H53	2					MF537244
H54	3					MF537245
H55	1					MF537246
H56		1				MF537247
H57		1				MF537248

Figure S1. A visual representation of the scenarios tested for each of the questions asked [illegible] carrying out our ABC analyses. The populations in question are located at the center of ea[illegible] while the possible sources surround it. Each of the surrounding boxes represents a unique scenario that was modeled, with exception to distinct native populations (i.e. CN, JP, and [illegible] that also had every possible admixture option modeled as well, and the boxes connected b[illegible] dashed red arrow indicating another alternative scenario. The two letter abbreviations wer[illegible] for each country, with the lowercase letters "s", "w", and "e" representing the cardinal dir[illegible] of south, west, and east respectively. The abbreviation "Nat" indicates native range, mean[illegible] scenarios for what the native source could be were tested prior to continuing with the disp[illegible] question.

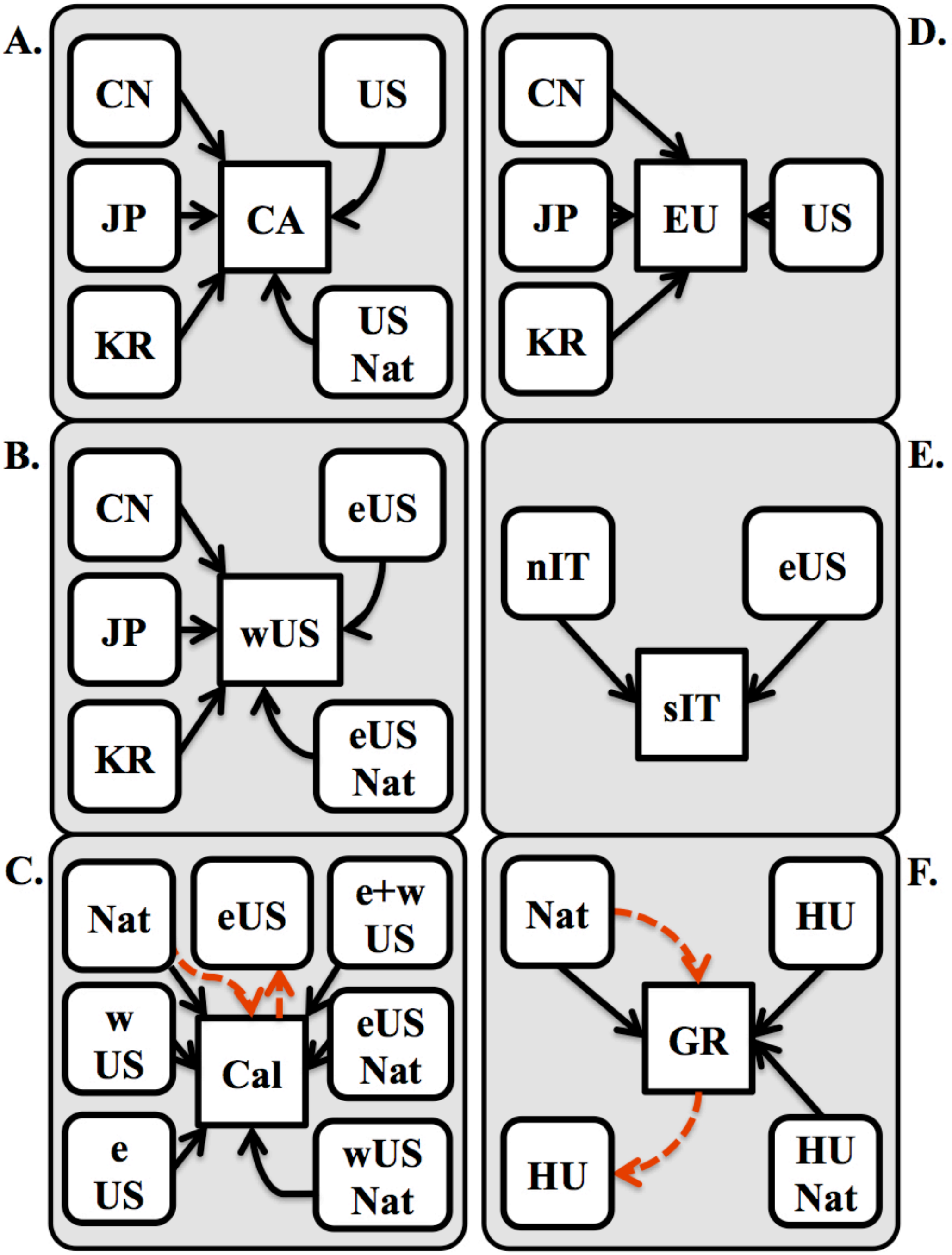

Supplement: Supplementary file 1 — Supplementary Table and Figure [file 41598_2017_10315_MOESM1_ESM.pdf]
